# Navigating Black Aging: The Impact of Stress and the Power of Resilience in Promoting Health

**DOI:** 10.1093/geroni/igab046.370

**Published:** 2021-12-17

**Authors:** Sarah Forrester, Keith Whitfield, Catarina Kiefe, Roland Thorpe

**Affiliations:** 1 University of Massachusetts Chan Medical School, Worcester, Massachusetts, United States; 2 University of Nevada Las Vegas, Las Vegas, Nevada, United States; 3 University of Massachusetts Medical School, Worcester, Massachusetts, United States; 4 Johns Hopkins Bloomberg School of Public Health, Baltimore, Maryland, United States

## Abstract

The health profile of African Americans clearly shows that stress works to worsen chronic conditions. To improve the health of aging African Americans, interventions need to address how effects of stress are reduced by individual resilience factors and exacerbated by anxiety or other traits. We will characterize the effects of stress by measuring rate of biological aging (RBA) over thirty years in a Black cohort (aged 18-30 at baseline) of approximately 2,000 individuals from the longitudinal CARDIA study. Biological aging (BA) captures premature physiological aging beyond that predicted by an individual’s chronological age. RBA will be characterized by within person change in BA over 30 years. We will measure the association between RBA and anxiety and will further measure the extent to which various forms of individual resilience factors mitigate the effects of anxiety on BA. We will also explore how intersectionality is evinced in sex differences in RBA.

